# DEVELOPING COMMUNITY-BASED AGING SERVICES IN NORWAY: LESSONS FROM THE US

**DOI:** 10.1093/geroni/igae098.0193

**Published:** 2024-12-31

**Authors:** Andrew Scharlach, Laila Tingvold

**Affiliations:** University of California Berkeley, Alamo, California, United States; Norwegian University of Science and Technology, Trondheim, Sor-Trondelag, Norway

## Abstract

Ongoing transformations in the Norwegian welfare state reflect decreasing reliance on government and an increasing role for civil society. Norway’s current “Live Safely at Home” policy, for example, aims to expand community-based supports to enable older adults to live at home for as long as possible, while simultaneously promoting community engagement and social participation. In an effort to learn from the US’ well-established mix of governmental and non-governmental aging support services, we began a study of the experiences of community-based aging service providers in the US. In the first phase, we conducted six weeks of ethnographic fieldwork and 31 in-depth interviews with staff and participants at aging service provider organizations in Northern California. Data were analyzed following thematic analysis procedures proposed by Braun and Clarke (2006, 2019). An overarching theme emerging from the data was tension between the goals of personal support and social inclusion. Organizations focused primarily on community engagement (e.g., senior centers, Villages) appeared to have limited ability to serve individuals as their physical or cognitive functioning declined, while organizations focused primarily on personal care typically provided little attention to fostering community engagement or social participation. These findings reflect a tension between the goals of “aging-in-place” and “aging-in-community.” As Norway develops a more comprehensive community-based aging services system, it will be important to ground services in existing social institutions and informal social networks, enabling older Norwegians with functional disabilities to live safely at home while maintaining meaningful social and community engagement.

